# Participants' perspectives on improving retention in HIV care after hospitalization: A post-study qualitative investigation of the MAPPS study

**DOI:** 10.1371/journal.pone.0202917

**Published:** 2018-08-27

**Authors:** Sophie G. Minick, Sarah B. May, K. Rivet Amico, Jeffrey Cully, Jessica A. Davila, Michael A. Kallen, Thomas P. Giordano

**Affiliations:** 1 Department of Medicine, Baylor College of Medicine, Houston, TX, United States of America; 2 Center for Innovations in Quality, Effectiveness and Safety (IQuESt), Michael E. DeBakey VA Medical Center, Houston, TX, United States of America; 3 Department of Health Behavior and Health Education, School of Public Health, University of Michigan, Ann Arbor, MI, United States of America; 4 Department of Psychiatry and Behavioral Sciences, Baylor College of Medicine, Houston, TX, United States of America; 5 Department of Medical Social Sciences, Northwestern University Feinberg School of Medicine, Chicago, IL, United States of America; Johns Hopkins School of Public Health, UNITED STATES

## Abstract

Few interventions have been shown to improve retention in HIV care. We recently completed a randomized, controlled trial of a peer mentoring intervention, which failed to increase retention in care or HIV suppression. We sought to gain insight into this negative result and elicit suggestions for future interventions. We conducted semi-structured one-on-one interviews with a sub-sample of participants and all available interventionists after completion of the primary study. Interviews were coded by two researchers and thematically analyzed. Participants in the intervention arm (N = 16) reported good rapport with and benefit from peer mentoring and found the mentors helpful in facilitating the transition from hospital to out-patient clinic. Control arm participants (N = 9) reported similar emotional and social support benefits from the health educators. In both arms, ongoing challenges including completing paperwork, securing transportation, and rescheduling missed appointments were cited, along with internalized stigma and lack of will to seek care, despite the mentors’ best efforts. Suggested improvements to the intervention included: more frequent contact with interventionists; additional support for mental health problems; and targeting overall health rather than a more selective focus on HIV. Mentors and health educators agreed with the participant-reported barriers and added that some participants were too sick to meaningfully participate in the intervention, while others appeared unwilling to engage with the interventionists in a meaningful way. Mentoring was highly acceptable and felt to be impactful, however it was not sufficient to overcome structural barriers or stigma and low motivation in some participants. The attention control intervention may have had an unintended positive impact. Future interventions should focus on broad aspects of health and well-being.

## Introduction

Antiretroviral therapy (ART) for HIV infection results in sustained virus suppression in people living with HIV (PLWH), which leads to improved survival. PLWH must be retained in HIV care to maintain access to ART and undergo safety monitoring as well as benefit from treatment and prevention of comorbid conditions. Generally, in the US, PLWH should see a clinician every 3-to-6 months, and more frequently as needed. Retention in HIV care is a strong predictor of clinical outcomes, including viral load (VL) suppression and survival [1–3]. A small number of interventions explicitly designed to improve retention have been developed and tested in randomized trials. One study enrolled persons in the HIV clinic who, based on previous missed visits, were at risk for poor retention [4]. The intervention was based on trained lay staff members providing brief education and enhanced contact and resulted in small but meaningful increases in retention compared to usual care. Another recently completed study used contingency management, in which persons who completed laboratory tests on time were paid. It, too, yielded small improvements in retention [5].

Unlike clinic-based studies, where the patient is at least somewhat engaged in care, two recently completed studies focused on out-of-care patients who were found and recruited while hospitalized. Our Mentor Approach for Promoting Patients’ Self-Care (MAPPS) relied on peer mentoring, while the Hospital Visit as Opportunity for Prevention and Engagement for HIV-Infected Drug Users (Project HOPE) study tested both patient navigation and contingency management [6, 7]. Neither intervention resulted in sustained improvements in retention in care.

The MAPPS study was a prospective, randomized, controlled trial to examine whether a peer mentoring program targeting hospitalized people living with HIV (PLWH) who were either out of routine HIV care or recently diagnosed improved linkage and retention in HIV care [6]. Participants were recruited from Ben Taub Hospital, a publicly funded hospital that primarily cares for persons without insurance or with public insurance. Hospitalized patients who planned on seeking follow-up at Thomas Street Health Center, a free-standing comprehensive clinic for persons with HIV, were randomized into a mentoring arm or the control arm. To be eligible, patients could not be “in care,” which we defined as: having completed an HIV primary care visit at TSHC in at least three of the four previous quarter-years and having had at least three consecutive HIV VL results <400 c/mL over at least six months, the most recent of which was within three months of enrollment. All persons not “in care” were defined as “out of care,” including persons who were diagnosed with HIV infection for less than one year and patients who intended to transfer care to TSHC after discharge. In the mentoring arm, Thomas Street Health Center peer mentors living with HIV served as lay counselors and potentially role models for successfully managing HIV infection and engaging in active self-management. The intervention was based on the information, motivation, behavioral skills (IMB) model [8, 9]. Information was provided in the form of brochures and brief instruction focused around the importance of obtaining outpatient HIV care and how to navigate the health care system. Motivational factors relied on the mentors sharing their personal stories, many of which involved overcoming barriers to care, to build rapport, social support, and instill hope. Behavioral skills components centered on goal-setting and action-planning to increase care-seeking behavior. Action planning included the mentor facilitating a discussion in which the patient identified barriers to care and steps he or she could take to overcome those barriers. The peer mentors were instructed to use a semi-structured delivery that was facilitated with a checklist of topics. The checklist included telling their story to the patient, eliciting concerns about HIV from the patient, asking the patient to discuss the importance and confidence in obtaining outpatient HIV care, assessing barriers to care, conducting an action planning around overcoming those barriers, and providing informational brochures about the clinic and about treatment of HIV. The intervention was developed to be implemented in 2 in-person sessions before discharge and 5 phone calls in the 10 weeks after discharge from the hospital. The control group received an “attention” control designed to mimic the intensity of the mentoring intervention without providing content focused on retention in care. This control intervention was delivered on the same schedule as the MAPPS intervention but focused on providing education on safer sex and safer drug use for persons living with HIV to avoid transmission of HIV. The prevention intervention was based on Project RESPECT, which is based on the theory of reasoned action and social cognitive theory [10]. The control intervention contact time was comparable to that of MAPPS, including in-person and telephone sessions, but its tone was didactic and its content was educational, and did not involve peers. Paid health educators delivered the control intervention. All the interventionists were trained and underwent periodic fidelity assessments with standardized patients and used checklists to guide content delivery [11]. The checklists were submitted after each session and nearly all sessions addressed all topics. The trial was registered at ClinicalTrials.gov (NCT01103856).

The primary outcome in the MAPPS trial was a composite of both retention in care (attend at least 2 HIV primary care visits, one within 30 days of discharge and one between 31 and 180 days after discharge) and VL improvement (VL suppression [<400 c/mL] or improvement [≥1 log reduction] from hospital baseline VL. The MAPPS study successfully recruited and enrolled 417 out-of-care hospitalized patients with HIV between August 2010 and August 2013. Demographic and baseline clinical characteristics did not differ between the arms. Prospective follow-up for the primary outcome for the MAPPS study was completed in February of 2014.

Compared to the control intervention, the MAPPS intervention did not increase the composite outcome or its components of retention in outpatient HIV care or viral load improvement [6]. To gain additional knowledge from the MAPPS study, we designed and executed a follow-up qualitative study. The objectives of this qualitative study were to both obtain feedback to try to understand the negative MAPPS trial outcomes from the participants and interventionists themselves, as well as to gain information on how to move forward with the design of subsequent interventions to improve retention in HIV care.

## Methods

### Participants

**Fig 1:**
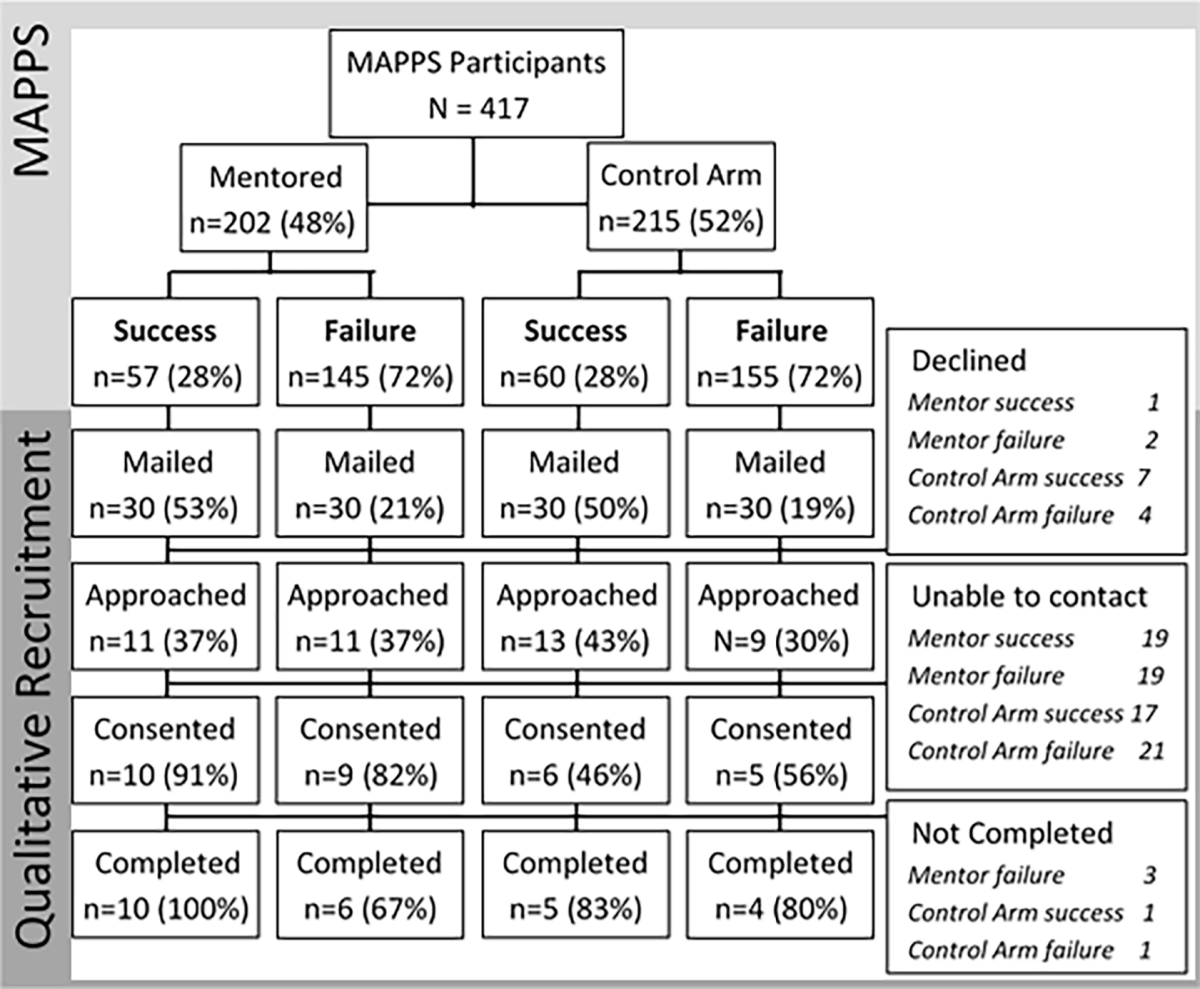
Participant flow.

Participants for this qualitative sub-study were recruited from February 2016 to April 2016 through purposefully sampling past MAPPS study participants. Equal numbers of participants were approached from the mentored and attention control arms of the MAPPS study according to outcome, i.e., success or failure as determined by joint VL and retention in care outcomes (Fig 1). Thus, four groups of participants were recruited: Mentored and “success,” Attention Control and “success,” Mentored and “failure,” and Attention Control and “failure.” Participants were considered ineligible for the sub-study and not contacted if they were not alive, not cognitively aware, in jail/prison, or in hospice care per medical record review. Eligible participants were approached chronologically, in batches of 20 (5 from each group), with those who completed the MAPPS study most recently approached first, until data saturation was reached. Participants were contacted via letters introducing the study and providing an option to opt-out of receiving recruitment phone calls. Potential participants who did not opt out were called at the last known phone number and invited to participate. To allow for telephone interviews, we requested and were granted a waiver of written informed consent for this qualitative sub-study from the IRB. Potential participants were read an informed consent document and asked if they were granting their consent to participate in the research; if so their verbal response was recorded on study logs. Mentors and health educators who delivered the interventions were recruited directly by the investigators, invited to participate in the sub-study, and also provided verbal informed consent. This sub-study was approved by the Institutional Review Board for Baylor College of Medicine and Affiliated Hospitals.

### Procedures

Interviews were conducted by trained research coordinators who took notes as well as recorded audio from the interview. Study participants were asked about their experience with their HIV care after they left the hospital where they originally enrolled in the MAPPS study. Interview questions and probes focused on whether the participants felt the interventions’ message and means by which this message was delivered by the interventionists (mentors or health educators) during the intervention made a difference in their desire and ability to stay in care; how the program should be adjusted in the future; and general suggestions for ways to improve linkage and retention in HIV primary care (Table 1). Interviews were in person (no participants elected for telephone interviews) and lasted no more than an hour, excluding time for consent and paperwork. Mentors and health educators who delivered the interventions were queried about challenges they faced delivering the study intervention and what areas they felt the intervention was successful or where it could be improved (Table 1).

**Table 1:** Major topics and key interview questions.

**Study participants**
About you and HIV care
Hospital experience and anything remembered 6 months post discharge relating to HIV care
What made it easier/ harder for you to see your HIV doctor and get your HIV under control?
How did you deal with these things? Did you get the support or help you needed?
What could be done differently for other patients discharged from hospital to get HIV care?
About MAPPS sessions
Did working with the mentor (health educator) help you get HIV care after discharge from the hospital?
Did the mentor (health educator) influence how you feel about living with HIV?
Most/ Least helpful aspects from mentor (health educator)?
Were there things about the visits that you would change or like to see improved?
Were there things about the phone calls that you would change or like to see improved?
**Interventionists**
About yourself
Why did you participate? Do it again?
Most/Least challenging about being an interventionist?
What can we do to make that less challenging or bothersome to you?
How did being an interventionist affect you?
About MAPPS sessions
Most / Least helpful aspects for participants
Patient needs unequipped to address
Barriers to patients participating
Suggestions for future interventions

### Analysis

Baseline demographic, socioeconomic and clinical characteristics reported by the MAPPS participants were abstracted from the MAPPS databases for the entire MAPPS study and the sub-set enrolled in the qualitative study. Statistical testing on these variables was not performed since we were not testing hypotheses. Regarding the qualitative data, narrative summaries were created for each interview using notes taken during interviews as well as notes made while listening to the audio recordings. Audio recordings were transcribed and transcripts were imported into Atlas ti. version 6.2 for data management. As data were collected, content analysis was performed and themes were extracted deductively. Content analysis allowed data to be summarized and categorized into themes and categories [12]. Recruitment ceased once data saturation was reached in each stratum. Two researchers, SBM and SGM developed a list of codes initially corresponding with the topics of the interview questions. Using these codes, the researchers individually analyzed transcripts from the initial interviews. More codes were created to represent new data as needed. The code lists were then harmonized and a code book was developed. Using these codes researchers analyzed subsequent interviews. If passages were not similarly coded by both researchers, they were presented to other members of the study team and a consensus was reached. All transcripts and codes were compared and validated by SBM, SGM, and TPG. Separate though overlapping codes were created for analyzing mentor and health educator interviews. Thematic analysis proceeded to present data cohesively.

## Results

### Participant characteristics

Twenty-five of the original 417 MAPPS participants were interviewed (Fig 1). Eight of the 11 interventionists (5 mentors, 3 health educators) were also interviewed for their first-hand experience in administering the mentor and the attention control interventions. A total of 33 interviews were conducted, transcribed and thematically analyzed. Baseline demographic, socioeconomic, and clinical characteristics of the sub-set of participants in the qualitative study were similar to those of the full MAPPS sample, though persons who reported substance use and more unmet needs were less well represented in the qualitative study (Table 2). The 25 participants included 88% and 56% who had VL improvement and retention in care at 6 months, respectively, and 80% and 92% who achieved VL <400 c/mL and retention in care at 12 months, respectively.

**Table 2:** Demographic, socioeconomic and clinical characteristics at the time of enrollment in MAPPS of all MAPPS participants (N = 417), MAPPS participants who received an invitation to participate in the qualitative sub-study but did not (N = 95), and MAPPS participants who were invited to participate in and completed the qualitative sub-study (N = 25).

	**MAPPS Trial** **N = 417**	**Invited but Did Not Participate** **N = 95**	**Qualitative Sample** **N = 25**
Sex			
Male	305 (73%)	67 (71%)	18 (72%)
Female	112 (27%)	28 (29%)	7 (28%)
Race			
Black	278 (67%)	59 (62%)	20 (80%)
Hispanic	81 (19%)	17 (18%)	2 (8%)
White	58 (14%)	19 (20%)	3 (12%)
Age (years)			
<30	52 (13%)	16 (17%)	4 (16%)
30–39	114 (27%)	22 (23%)	7 (28%)
40–49	146 (35%)	37 (39%)	5 (20%)
≥50	105 (25%)	20 (21%)	9 (36%)
Sexual Identity			
Gay/Lesbian	117 (28%)	32 (34%)	8 (32%)
Heterosexual	255 (61%)	54 (57%)	14 (56%)
Bisexual	32 (8%)	6 (6%)	2 (8%)
Not sure/in transition	11 (3%)	3 (3%)	1 (4%)
Initial VL (copies/mL)			
< 400	176 (42%)	19 (20%)	8 (33%)
> 400 or missing	241 (58%)	76 (80%)	17 (67%)
Initial CD4 cell count (cells/mm^3^)			
< 200	269 (65%)	57 (60%)	14 (56%)
200–500	81 (20%)	14 (15%)	5 (20%)
> 500	65 (16%)	24 (25%)	6 (24%)
HIV diagnosis			
New this hospitalization	47 (11%)	13 (14%)	3 (12%)
Previous, <1 year since diagnosis	65 (16%)	13 (14%)	7 (28%)
> 1 year since diagnosis	306 (73%)	69 (73%)	15 (60%)
HIV Risk Factor			
Men who have sex with men (MSM)	146 (35%)	34 (36%)	10 (40%)
Injection drug use (IDU)	58 (14%)	13 (14%)	2 (8%)
Neither MSM or IDU	213 (51%)	48 (51%)	13 (52%)
Substance use in last 3 months			
Any drug use not including marijuana	111 (27%)	33 (35%)	2 (8%)
Marijuana only	61 (15%)	13 (14%)	5 (20%)
None	240 (58%)	48 (51%)	18 (72%)
Depression			
Not depressed: PHQ8 <10	223 (53%)	45 (47%)	15 (60%)
Depressed: PHQ8 ≥10	191 (46%)	50 (53%)	10 (40%)
Stigma			
Median score (25^th^ and 75^th^ percentile)	26 (22, 28)	25 (22, 29)	24 (23, 28)
Employment status			
Employed	86 (21%)	21 (22%)	9 (36%)
Not employed	329 (79%)	74 (78%)	16 (64%)
Housing Status			
Living in home you own or rent	246 (62%)	44 (51%)	13 (52%)
Living in home of friend or family member	129 (33%)	37 (43%)	11 (44%)
Living in halfway house, rehab, homeless shelter, on street	19 (5%)	6 (7%)	1 (4%)
Insurance			
Private insurance, Medicare or Medicaid	123 (29%)	30 (32%)	4 (16%)
Harris Health System enrollee	172 (41%)	44 (46%)	13 (52%)
No Insurance, not Harris Health System enrollee	115 (28%)	21 (22%)	8 (32%)
Unmet Needs			
Median number (25^th^ and 75^th^ percentile)	3 (1, 6)	4 (1, 6)	1 (0, 4)

### Qualitative results

Thematic analysis revealed that the themes that emerged were similar across primary study outcomes (success or failure), randomized arm (mentored or control), and interventionist role (peer mentor or health educator). Results are presented by role in the study.

#### Mentored participants

Mentored participants found working with the peer mentor to be very helpful, particularly in providing a healthy framework for dealing with HIV. They described mentors to be encouraging and supportive, like talking to a friend or family member rather than medical staff.

“You know Ms. X (mentor) is not like you’re speaking to a healthcare official or anything like that. It’s like a friend… she just seems to be just so sympathetic and empathetic to our needs and your issues.” -47 yr old Hispanic male, neither retained nor VL improved at 6 months (mentored)

The peer mentor was said to also be a source of motivation for the participants to take better care of their health and ease the transition from hospital to the clinic.

“Yeah so it was in the head thing and she got in there and see and swept all the cob webs out and said, “No, no um-um; it ain’t going to be like that. You stand strong. You’re a survivor.”– 46 yr old Black male, retained and VL improved at 6 months (mentored)

Mentored participants found follow-up phone calls from their mentor post-discharge to be helpful to keep them on track with their HIV care, by both providing emotional support and guidance.

“It makes your transition to where you’re going much easier. You trust that mentor. That mentor will take you wherever you have to go and walk you through it and not just leave you there.”—50 yr old Black female, retained and VL improved at 6 months (mentored)

The qualitative data from the mentored participants suggest that the intervention did provide support and improve understanding and motivation, as intended.

#### Attention control participants

Similarly, patients in the attention control arm reported increased motivation and support. They discussed developing a strong rapport with the health educators, noting the mental and social support received from their health educator.

“Yeah, he’d call me all the time. He called me like once a week. It was nice that he was concerned or that somebody was concerned to make sure that I was still staying healthy.” -35 yr old Black male, not retained but VL improved at 6 months (attention control)

They liked the phone calls they received from the health educator checking up on them, and, despite the protocol’s attempts to keep the phone calls focused on education about the prevention of transmission of HIV, the participants in the control arm viewed the interventionists as motivational and an “open ear” that they could talk to.

“… she did those things to make me come back to life and be like hey, your life is too precious to give it up now. So yeah, I liked speaking with her. It was very encouraging. It was very powerful.” -21 yr old Black male, retained and VL improved at 6 months (attention control)

“Yeah, me and him were real close, and he’d give me good advice. He was a big help for me too.” -32 yr old Black male, neither retained nor VL improved at 6 months (attention control)

#### Interventionists

The interventionists from the study interacted with the patients either in the role of a peer mentor with HIV (intervention arm) or as a health educator (attention control arm).

Peer mentors all reported participating in MAPPS as a rewarding experience. They felt it allowed them to empathize with the participants and help them overcome misconceptions. It also solidified their identity as a mentor and leader among patients.

“But there were a lot of people there that were ready to give up, and then when they saw me, I guess my age, when they saw me, I told them don’t give up. There is a way. You know? There is a way, and I would talk to them, and they would be like you’re holding my hand. I said of course I am. I’m holding your hand. I’m the same like you. I’m HIV.”–Peer mentor

However, mentors wanted more time with patients and reported that in some cases the patient may have be too physically sick or psychologically unwilling to benefit from their interactions.

“There were some challenges because there were some that no matter what you said to them, they were still not going to adhere.”–Peer mentor

Likewise, health educators built good rapport with patients and found it difficult not to get involved even though it was beyond their defined role as the control arm.

“I’m also a people person, so I get attached to you, and I’m not supposed to.”- Health educator

Like the mentors, health educators also found some patients too sick to engage in the intervention.

“Sometimes I think how sick they were really affected what we could actually do.”–Health educator

In addition, they noted that some aspects of the health education were uncomfortable as the information may have been less relevant to patient’s condition.

“They had multiple complicated infections, and I just didn’t quite understand why I needed to talk to them about viral load and condoms when they were so incapacitated. I think the relevance of that level of information was very general, and it was very basic, and these people did not have basic infections.”–Health educator

#### Suggestions for improving reengagement in care and interventions

While some participants in the study did re-engage in care and had improved viral load, there were no differences in these outcomes between the study arms, suggesting that there were barriers that the peer mentor intervention did not address more effectively than the attention control intervention. Some of these factors may not be within the control of the individual but rather are broader structural or community factors including social support, transportation, scheduling appointments, and paperwork.

“It’s just such a long wait time. I missed my last appointment, and they can't even get me in until (3 months later) now.”- 51 yr old White male, retained and VL improved at 6 months (mentored)

In contrast, some factors are within the participant’s control and are less readily influenced by others. These include mindset, will, readiness to change, and valuing health.

“I mean, see it’s all about the patient and whether they want the help or they don’t want the help.” - 33 yr old Black male, retained and VL improved at 6 months (mentored)

Even a participant who did not meet the primary study’s definition of success recognized the participant’s critical role in re-engaging in HIV care and the primacy of motivation over structural barriers.

“These people make appointments with you, but you have to make the appointment. You’re the one that has to show up. They’re here to help you. So you have to make the step to help yourself and get here and get the help. You have the will to choose to do or not to do. So it’s up to you.” - 44 yr old Black male, neither retained nor VL improved at 6 months (attention control)

Fear and stigma of being recognized at the HIV clinic and having their status revealed were described by participants as barriers to HIV care.

“It (the clinic) is for people that you know that everybody just have HIV. So at first it was like man, I don’t wanna go here. Do you know what I’m saying? -32 yr old Black male, neither retained nor VL improved at 6 months (attention control).

We probed for detailed suggestions on how to improve motivation and change behavior, but not surprisingly participants were not able to identify solutions beyond mentoring.

Several participants reiterated the need for an accepting community and suggested this acceptance may be brought about with support groups, public education, integrating friends and family and encouraging peer interaction. Participants would like the interventionist to be present in a positive way. Many participants concluded that others, and peers in particular, could help a person be mentally stronger, though ultimately a person makes his or her own decisions.

“I really want to share with some people really because I see some friends you know who have the same disease but ashamed to you know to go out and tell people… You guys share each other maybe or maybe care for each other; maybe it can be a better place.”- 38 yr old Black male, retained & VL improved at 6 months (mentored)

“You know yeah because some people need a push a little bit… push should come from like people like us.”– 38 yr old Black male, retained and VL improved at 6 months (mentored)

Participants wanted more time with the mentor to work to increase motivation and decrease barriers.

“Come in and talk to the patient. You know daily.” - 46 yr old Black male, retained and VL suppressed/improved at 6 months (mentored)

Participants recognized that an intervention would need to address issues beyond HIV and role modeling what living successfully with HIV could look like. They suggested that an intervention include components that a peer could not deliver.

“I would say something more structured, thinking more um transportation. Housing or paperwork you know just for small things and when you get out of the hospital; do you have a place to go? You know when you um and do you have you know access to food blah blah blah, just the basics… They’re there (at the hospital). That’s when it’s really easier for you to check off and go through and see what their basic needs and assess all of that and see what you can and what yall cannot do to help them out… Making sure that they fully understand the benefits and everything that that’s there to offer for them… if you had it all in one spot and that would be awesome.”– 47 year old Hispanic man not retained nor VL improved at 6 months (attention control)

Because most patients might be just be sitting up there and they’re so frustrated and stressed out… And no one will listen yeah. They’re getting you care but they’re not actually listening to what’s really going on with you…. You’re already going through a a health issue. Now you’re going through a mental issue too.– 46 yr old Black male, retained and VL improved at 6 months (mentored)

“Counseling. Like from a professional. That’s what you need because it’s a lot of people that are in denial today. Like they’re in so much denial that they done let themselves down”– 32 yr old Black male not retained nor VL improved at 6 months

Interventionists working with the participants also recognized the need for a more extensive and comprehensive interaction involving more time and addressing needs other than HIV. They also felt other social services approaches may have added benefit in reengaging people in care.

“It’s nothing but 2 visits. I would love to visit them sometimes more. But it was one and then the next day. Then, sometimes you didn’t get but one visit because they go home.”- Peer mentor

“Because they’re having all kinds of crises in their life that aren’t just the HIV thing… Is there a way to get them in touch with somebody who can help them with some of these other social problems that they’re having?”–Health educator

## Discussion

We previously conducted a randomized controlled trial of a peer mentor intervention called MAPPS to improve retention in care and HIV viral load in persons hospitalized and out of HIV care. Despite preliminary data from outpatients and pilot data from inpatients suggesting that the mentoring intervention could improve retention in care, the MAPPS intervention did not result in significant improvements in those outcomes compared to an active attention control, with 28% of participants in each group meeting the composite endpoint of success at 6 months (retention in care and VL<400 c/mL) [6]. The current sub-study conducted qualitative interviews with participants and both the peer mentors and control interventionists from that study to try to understand these negative results and gain insight into what subsequent interventions could do differently. The results suggest ways for the field to move forward.

First, the peer mentoring intervention may not have been potent enough to overcome some external barriers to care including logistical and structural barriers, e.g., paperwork, scheduling issues, and transportation. Peer mentoring was extremely well received and the peers were trained to inform patients about available services, however they were not navigators, social workers, or case managers. Most of the patients in both arms of the study met with a service linkage worker as standard of care, but some participants in the present study clearly reported the need for additional services to address their unmet needs. Nevertheless, a recently completed trial failed to demonstrate sustained benefit of navigation services in hospitalized, out-of-care PLWH [7]. These barriers may be amenable to provision of additional navigation and social services, but navigation alone does not appear sufficient. It is also possible that structural barriers were cited when in fact the underlying problem was motivational or related to stigma. A reasonable next step would be to intervene with a combination of peer mentoring and navigation services.

Second, there may be some barriers to care such as motivation, readiness to receive HIV care, stigma, mental health, and substance use conditions that peers and navigators were unable to address and for which a more comprehensive navigation and wellness approach may be better suited. Recent applications of the IMB model to engagement in care situate information, motivation and skills within a socioecological framework, suggesting that policy, structural, inter- and intra-personal factors influencing the delivery and receipt of care need to be broadly incorporated into intervention programs [13]. Participant discourse suggests that specific attention to factors contextualizing available care and experiences accessing it are indeed important. While peers did discuss access and mental health issues with participants, participants may have needed a greater emphasis on these critical factors than the peers could reasonably provide. Provision of non-urgent mental health and counseling services to increase motivation, screen for and address depression and substance use, and promote self-care action could be studied. However, such services are not routinely offered in the acute care setting and could add substantial cost to care since they require trained clinicians and could increase length of hospital stay. Motivational interviewing techniques have been used with some success to improve adherence to ART and could also be studied in this population [14]. Designing and packaging such a multi-faceted intervention for sustained delivery would be challenging.

Third, participants and interventionists noted that some patients needed care for medical issues beyond HIV. If the participant does not rate HIV care as their greatest unmet health need, then a strategy promoting HIV care may not be salient. While Thomas Street Health Center does provide comprehensive medical care, the range of other services provided may not have been emphasized enough by the interventionists. Partnering with other agencies to help address unmet needs outside of HIV care, including housing, food, and employment, could have strengthened our intervention but would be challenging to initiate in the hospital. Future interventions could be more oriented to the health benefits of comprehensive care and supportive services that include rather than focus on HIV care.

Fourth, some hospitalized patients were too sick to fully participate in the intervention and get its full benefit. To some degree this limitation is inevitable in a study of hospitalized persons. It does suggest the need to include post-discharge intervention components (e.g., phone calls as in MAPPS).

Finally, it is very likely that the control intervention did in fact provide support that benefited participants. We attempted to distinguish the peer and education interventions in many ways. The peers were PLWH and patients who wore badges identifying them as volunteers, while the educators were paid staff who were generally younger and had education training. The peers were instructed to be conversational and use narrative, while the educators were didactic in their approach. The peers focused on the participant’s HIV health, which the educators focused on avoiding forward transmission of HIV. The educators were also instructed to refer participants with questions and needs to the clinic. Nonetheless, the results of the present study clearly indicate that the participants in the control arm did derive support from the educators. It is impossible to quantify the benefit of the attention control intervention since there was no “usual care” group. Since providing attention and support was an active ingredient of the peer intervention, we suggest that subsequent trials of interventions focused on mentoring and support should use a “usual care” control group rather than an “attention” control group. An alternative but more complex design would include three arms: the intervention arm, an attention control arm, and a usual care arm. Proving that a relatively simple intervention is better than usual care may be sufficient to justify adoption, while more complex and/or costly interventions might need to be compared against an attention control. In retrospect, the MAPPS intervention probably could have been compared against usual care with meaningful results. Study design considerations aside, the benefit that participants from both arms of the study reported from the interventionists suggest that this highly vulnerable population of PLWH who are hospitalized and out of care can benefit, at least qualitatively, from additional support.

This qualitative study has limitations that should be noted. First, there was no Spanish speaking interviewer in this sub-study to gain the perspectives of the Spanish-only speaking population. Second, between 2 and 3 years elapsed from when the participant completed the intervention to when the qualitative interviews occurred. This time gap may have made it difficult for participants to accurately recall the specifics of the intervention and the events that took place during and immediately after the hospitalization in which they were enrolled. Additionally, narratives may have shifted over the years with new experiences and newly acquired perspectives. Many of the participants who were interviewed are currently in care although they may not have been when the MAPPS study concluded, potentially introducing bias to the results. These participants who eventually succeed in getting into care could be more likely to attribute their success to personal attributes, such as mindset or will power, while patients who are still struggling could be more inclined to attribute their struggles to external causes. Finally, while we attempted to recruit a representative sample of participants, the interviewed participants appear to underrepresent people who report substance use and persons with more unmet needs, which may have influenced our results. We recommend that studies include qualitative methods in the original study design to gather participant feedback in a timely manner.

In conclusion, peer mentoring was perceived as impactful and highly acceptable to out-of-care hospitalized PLWH but likely did not provide enough structural support and was not broad enough to address the many and diverse barriers to HIV care that patients experience. The attention control intervention may have had an unintended positive impact on participants. The results of this research suggest directions for interventions that should be developed and tested in future studies.
